# Effect of Molybdenum on Plant Physiology and Cadmium Uptake and Translocation in Rape (*Brassica napus* L.) under Different Levels of Cadmium Stress

**DOI:** 10.3390/ijerph17072355

**Published:** 2020-03-31

**Authors:** Zhangxiong Han, Xuan Wei, Dejun Wan, Wenxiang He, Xijie Wang, Ying Xiong

**Affiliations:** 1 College of Environment and Resource, Northwest A&F University, Key Laboratory of Plant Nutrition and Agro-environment in Northwest China, Ministry of Agriculture, Yangling 712100, Shaanxi, China; han10260@163.com; 2 Testing and Quality Supervision Center for Geological and Mineral Products, The Ministry of Land and Resource, Xi’an 710054, Shaanxi, China; sxyljxhzx@163.com (X.W.); han10260@126.com (Y.X.); 3 Northwest Institute of Eco-Environment and Resources, Chinese Academy of Sciences, Lanzhou 730000, China; wei_liu_xuan@yeah.net; 4 Institute of Hydrogeology and Environmental Geology, Chinese Academy of Geological Sciences, Shijiazhuang 050061, China; wandejun@mail.cgs.gov.cn

**Keywords:** exogenous molybdenum, physiological parameters, plant biomass, chlorophyll fluorescence, cadmium uptake

## Abstract

This study investigated the beneficial effect of molybdenum (Mo) application on rape plants (*Brassica napus* L.) grown in a soil polluted by cadmium (Cd). A pot experiment was conducted to determine how different concentrations of exogenous Mo (0, 50, 100, and 200 mg/kg) affect plant physiology, biomass, photosynthesis, cation uptake, and Cd translocation and enrichment in rape plants under Cd stress (0.5 and 6.0 mg/kg). Under single Cd treatment, plant physiological and biochemical parameters, biomass parameters, leaf chlorophyll fluorescence parameters, and macroelement uptake of rape plants decreased, while their malonaldehyde content, proline content, non-photochemical quenching coefficient, and Cd uptake significantly increased, compared to those of the control group (*p*-values < 0.05). High-Cd treatment resulted in much larger changes in these parameters than low-Cd treatment. Following Mo application, the accumulation of malondialdehyde and proline decreased in the leaves of Cd-stressed plants; reversely, the contents of soluble protein, soluble sugar, and chlorophyll, and the activities of superoxide dismutase and glutathione peroxidase, all increased compared to those of single Cd treatment (*p*-values < 0.05). Exogenous Mo application promoted shoot and root growth of Cd-stressed plants in terms of their length, fresh weight, and dry weight. The negative effect of Cd stress on leaf chlorophyll fluorescence was substantially mitigated by applying Mo. Exogenous Mo also improved the uptake of inorganic cations, especially potassium (K^+^), in Cd-stressed plants. After Mo application, Cd uptake and accumulation were inhibited and Cd tolerance was enhanced, but Cd translocation was less affected in Cd-stressed plants. The mitigation effect of Mo on Cd stress in rape was achieved through the immobilization of soil Cd to reduce plant uptake, and improvement of plant physiological properties to enhance Cd tolerance. In conclusion, exogenous Mo can effectively reduce Cd toxicity to rape and the optimal Mo concentration was 100 mg/kg under the experimental conditions.

## 1. Introduction

Soil pollution by heavy metals is the main environmental problem arising from metal mines. Although the ecological governance of the main mineral elements is improved during the exploitation process, soil pollution by associated elements that are insufficient for metallogenesis is covert. Beneficial elements can modulate the uptake and translocation of such polluting elements in plants, causing changes in plant physiology and morphology, based on studies involving soil pollution by lead (Pb), cadmium (Cd), arsenic (As), chromium (Cr), and copper (Cu) [1,2,3,4,5].

Molybdenum (Mo) is an essential beneficial trace element for humans, animals, and plants [6,7]. China has abundant Mo resources. Molybdenum ores contain a low content of Mo, generally no more than 0.5%, and Mo is often associated with many valuable elements, such as Pb, Cd, As, Cu, iron (Fe), tungsten (W), gold (Au), and silver (Ag) [8]. Cadmium is the main polluting element associated with Mo ores, and its pollution characteristics and interactions with Pb, zinc (Zn), and selenium (Se) have been studied in plants, such as rice (*Oryza sativa* L.), wheat (*Triticum aestivum* L.), and rape (*Brassica napus* L.) [9,10,11,12,13]. It is believed that the harm of Cd to plants is mainly attributed to its available form that enters the plant [14].

In the plant, Cd is primarily bound to cell walls and less distributed in chloroplasts and mitochondria. Therefore, Cd stress is mainly manifested in direct damage of the plant, which in turn decreases plant physiological performance, biomass yield, and nutrient uptake and utilization [15]. How Cd induces stress in plant photosynthetic and physiological processes is still poorly understood, and the possible influencing factors include stomatal and non-stomatal factors. It is generally thought that Cd stress is due to the comprehensive effect of Cd on chlorophyll content, photorespiration process, ribulose-1,5-bisphosphate carboxylase/oxygenase (Rubisco) activity, ribulose-1,5-bisphosphate (RuBP) regeneration, photochemical efficiency of photosystem II (PSII), and Mehler’s reaction in the plant [16].

The mechanisms of Cd stress on rape have been reported in some studies. Evidence suggests that the subcellular accumulation of Cd is substantially altered in the leaves of rape under long-term Cd induction. Additionally, Cd stress exhibits a pronounced effect on the antioxidant system and cell structure in rape seeds during germination [17,18]. Under Cd stress, the structure of root tip cell walls of rape is susceptible to Cd-induced damage, which in turn shortens the roots [19] and inhibits the plant growth of rape. Specifically, leaf chlorophyll content is reduced and leaf photosynthetic capacity is decreased [20], while the antioxidant system is destroyed and hormone levels are imbalanced [21,22], leading to severe decline in the biomass and yield of rape [23]. The effect of Cd on rape plants has also been evaluated under the application of beneficial elements, such as Se and Zn [24,25]. Considering Mo, studies have explored its uptake in rape and involvement in the synthesis of nitrate invertase [26,27,28]. However, no study has reported on the mitigation effect of Mo on Cd stress in rape.

In this study, a pot experiment was conducted using rape plants grown in Cd-polluted soil conditions. Different concentrations of exogenous Mo were added to the soil to evaluate the effect of Mo on plant growth, physiological properties, and Cd uptake and translocation in Cd-stressed rape. The objectives of the study were to explore the mechanisms by which Mo mitigates Cd toxicity and find the optimal Mo concentration for application to rape plants. This study could provide useful information for sustainable agricultural production and environmental management in Cd-polluted soils around Mo mining areas.

## 2. Materials and Methods

### 2.1. Experimental Materials

The experimental soil was collected from farmland near the Mo mining area of Huanglongpu (34°22′ N and 110°01′ E) in Luonan County, Shaanxi Province, China. The topsoil (0–20 cm) was obtained by multi-point sampling and thoroughly mixed before use. The soil was classified as yellow brown soil (Eutric Planosols), with a pH of 7.13 and cation exchange capacity of 13.2 cmol·kg^−1^. It contained 15.2 g·kg^−1^ of organic matter and 857 g·kg^−1^ of total nitrogen. The total Mo and Cd contents were 0.35 and 0.24 mg·kg^−1^, respectively. The soil was air-dried and passed through a 4.0 mm sieve.

The plant material used in the experiment was *Brassica napus* L. cv. Zaqin No. 1, a common rape cultivar in the local area. Seeds were from Huanglongpu Seed Sales Center (Luonan, Shaanxi, China).

### 2.2. Experimental Design

The study was conducted in an experimental field of the Shaanxi Experimental Institute of Geology and Mineral Recourses (Xi’an, China). The pot experiment was designed based on the range of Mo and Cd contents in typical Mo mining areas. A total of 12 treatments were performed (Table 1), with four replications per treatment. Exogenous Mo and Cd were applied by spraying aqueous solutions of cadmium chloride (CdCl_2_, Analytical Reagent) and ammonium molybdate (NH_4_MoO_3_, Analytical Reagent), separately and uniformly, to the experimental soil. The reagents were thoroughly mixed with the soil by stirring, and the mixture was filled into polyethylene plastic pots (40 cm × 20 cm × 20 cm), with 20 kg per pot. The pots were placed in the experimental field and equilibrated for at least 2 weeks.

Rape seeds (*Brassica napus* L. cv. Zaqin No. 1) were disinfected with 0.01% H_2_O_2_ for 4 h and then sown in pots (20 seeds per pot). After emergence, the seedlings were thinned in a timely manner and those with best growth performance were retained (six plants per pot). During the experimental period, deionized water was used to irrigate the pots from the base, and soil moisture content was adjusted according to weather conditions. Soil pH was adjusted between 6.5 and 7.5 every 2 days to maintain consistency among all treatments. A potassium-free 1/2 Hoagland’s nutrient solution was supplied every 7 days (500 mL per pot). Samples were taken 80 days after emergence.

### 2.3. Plant Sampling and Analysis

Whole plants of rape were collected from each pot of different treatments and replications. The plant samples were rinsed with tap water and then soaked in a 0.02 mol/L EDTA-2Na solution for 15–20 min, followed by a rinse with deionized water.

#### 2.3.1. Measurement of Chlorophyll Fluorescence Parameters

Leaf chlorophyll fluorescence parameters were measured the night before and on the day of sampling using a portable spectrometer (PAM-2500; WALZ, Effeltrich, Germany). First, initial fluorescence yield (Fo), maximum fluorescence yield (Fm), potential activity of PSII (Fv/Fo), and potential photosynthetic quantum yield of PSII (Fv/Fm) were measured when the leaves were fully dark-adapted at the night before sampling (3 h after nightfall, 22:00). Then, quantum yield (yield) and apparent electron transport rate (ETR) were measured when the leaves were fully light-adapted on the next day (13:00). In each treatment, the parameters were measured on the second leaf (from outside to inside) in four plants per pot. The instrument parameters and measurement methods were consistent with those used by Yaryura et al. [29]. The photochemical quenching coefficient (qP) and non-photochemical quenching coefficient (NPQ) were calculated using the following equations:

qP = (Fm’ − Fs)/Fv’ = 1 − (Fs − Fo’)/(Fm’ − Fo’)(1)

NPQ = (Fm − Fm’)/Fm’ = Fm/Fm’ − 1(2)

where Fm’ is the actual maximum fluorescence, Fs is the steady-state fluorescence, Fv’ is the actual variable fluorescence, Fo’ is the initial fluorescence when the action light is turned off, and Fm is the maximum fluorescence.

#### 2.3.2. Measurement of Physiological Parameters and Biomass

The following parameters were measured in the leaves of rape plants. Chlorophyll content was measured by ethanol-acetone extraction [30]. Malondialdehyde (MDA) content was measured by two-component spectrophotometry [31]. Free proline content was measured by acidic ninhydrin colorimetric assay [32]. Soluble protein content was measured by Coomassie brilliant blue colorimetric assay [33]. Soluble sugar content was measured by anthrone colorimetric assay [34]. Superoxide dismutase (SOD) activity was measured by nitroblue tetrazolium assay [35]. Glutathione peroxidase (GPX) activity was measured by guaiacol assay [36].

Plant length, fresh weight, and dry weight were measured in the shoots and roots separately. After rinse, the plants were dried with absorbent paper. A ruler was used to measure the lengths of the shoot and the root. Then the plants were divided into shoots and roots. The fresh weight of each part was weighed on an electronic balance (accuracy 0.001 g). The shoots and roots were oven-dried separately, at 105 °C for 6 h until a constant weight was reached. The dry weight of each part was measured. The measurement was repeated four times for each treatment.

#### 2.3.3. Quantification of Inorganic Cations and Heavy Metals

Inorganic cations and heavy metals in plant samples were quantified after pretreatment by microwave digestion (ETHOS A, MILESTONE, Sorisole, Italy). Briefly, Cd analysis was performed on the roots and shoots using an inductively coupled plasma-mass spectrometer (X Series 2; Thermo Electron Corp., Boston, MA, USA) [37]. Potassium (K^+^), sodium (Na^+^), calcium (Ca^2+^), and magnesium (Mg^2+^) ions were analyzed for whole plants using an inductively coupled plasma-atomic emission spectrometer (iCP6300; Thermo Electron Corp., Boston, MA, USA) [38].

### 2.4. Data Analysis

Bioconcentration factor: the ratio of Cd content in the plants and Cd content in the soil, which represents the enrichment capacity of plants for Cd [39]. Transfer coefficient: the ratio of Cd content in plant shoots and Cd content in plant roots, which represents the capacity of Cd to be translocated from the roots to the stems and leaves [40]. Tolerance index: the ratio of mean root length in Cd-stressed plants and mean root length in control plants (100%) [41].

One-way analysis of variance (ANOVA) was performed using IMB SPSS Statistics 23.0 (IBM Corp., Armonk, NY, USA). For multiple comparisons, the least significant difference (LSD) test was used with a significance level of 0.05. Data processing and figure drawings were performed using Microsoft Excel 2016 (Microsoft Corp., Redmond, WA, USA).

## 3. Results

### 3.1. Effects of Mo on Plant Physiological Parameters and Biomass in Cd-Stressed Rape

#### 3.1.1. Fresh and Dry Weights of Shoots and Roots

**Figure 1:**
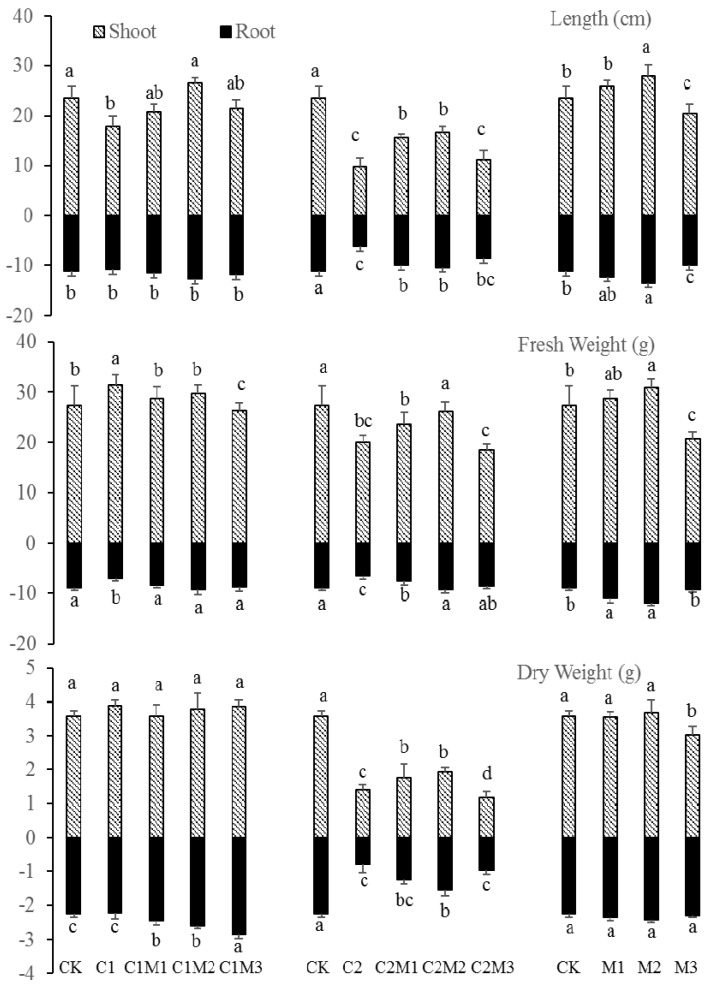
The biomass of rape plants with the co-treatment of Mo and Cd (*n* = 4). **Note:** Data are the mean ± standard deviation. Different lowercase letters above or below the error bars indicate significant differences (*p* < 0.05). CK (control group), C1 (treatment by 0.5 mg/kg Cd), C1M1 (treatment by 0.5 mg/kg Cd and 50 mg/kg Mo), C1M2 (treatment by 0.5 mg/kg Cd and 100 mg/kg Mo), C1M3 (treatment by 0.5 mg/kg Cd and 200 mg/kg Mo), C2 (treatment by 6.0 mg/kg Cd), C2M1 (treatment by 6.0 mg/kg Cd and 50 mg/kg Mo), C2M2 (treatment by 6.0 mg/kg Cd and 100 mg/kg Mo), C2M3 (treatment by 6.0 mg/kg Cd and 200 mg/kg Mo), M1 (treatment by 50 mg/kg Mo), M2 (treatment by 100 mg/kg Mo), M3 (treatment by 200 mg/kg Mo).

The changes in plant biomass under different treatments are shown in Figure 1. When the plants were treated with 0.5 mg/kg of Cd only (C1), shoot length and root fresh weight decreased compared to those of the CK group (*p* < 0.05). Following combined Mo application, both parameter values increased in groups C1M1–C1M3 compared to C1 (1.49- and 1.33-fold, respectively; *p* < 0.05). By contrast, shoot fresh weight increased in C1 compared to CK (*p* < 0.05), and decreased with increasing Mo concentration (*p* < 0.05). There were no significant changes in shoot dry weight, root length, and root dry weight between CK and C1 groups; however, root dry weight increased with increasing Mo application (1.29-fold; *p* < 0.05). These results indicate that 0.5 mg/kg Cd treatment reduced shoot length and root fresh weight while increasing shoot fresh weight in rape plants; exogenous Mo application promoted the growth of shoot length and root dry weight in rape plants under the low level of Cd.

When the plants were treated with 6.0 mg/kg of Cd only, the length, dry weight, and fresh weight of shoots and roots all decreased in the C2 group compared to those of the CK group (*p* < 0.05). Combined Mo application (C2M1–C2M3) resulted in substantial increases in plant length and weight compared to the C2 group (*p* < 0.05). The largest increases were recorded in the C2M2 group with 100 mg/kg Mo, yet the parameter values were still lower than in the CK group. These results indicate that 6.0 mg/kg Cd treatment considerably reduced the length and weight of rape plants, while the application of 100 mg/kg of Mo increased plant biomass under the high level of Cd.

When 100 mg/kg of Mo was applied alone, the length and fresh weight of shoots and roots increased in the M2 group compared to the CK group (*p* < 0.05). However, applying 200 mg/kg of Mo resulted in large decreases in shoot length, shoot fresh weight, and root length in the M3 group compared to the CK group (*p* < 0.05). These results indicate that 100 mg/kg of Mo had a positive effect on plant biomass in rape, but 200 mg/kg of Mo negatively affected it.

#### 3.1.2. Physiological Parameters

Significant changes were found in the physiological parameters in the leaves of Cd-stressed plants with or without Mo application (Table 2). When the plants were treated with 0.5 mg/kg of Cd, their MDA and proline contents increased in the C1 group compared to the CK group, and decreased in C1M1–C1M3 groups with increasing Mo concentration (35% and 19%, respectively; *p* < 0.05). Conversely, soluble protein and chlorophyll contents, as well as SOD and GPX activities, decreased in the C1 group and increased in C1M1–C1M3 groups (1.18-, 1.21-, 1.10-, and 1.21-fold, respectively; *p* < 0.05). Soluble sugar contents decreased in all the four groups compared to the CK group, but the effect of Mo was not significant. Following combined Mo application, SOD activity was enhanced, along with increased proline and decreased soluble sugar contents compared to the CK group. The contents of MDA, soluble proteins, and chlorophyll, and GPX activity were generally maintained at similar levels compared to the CK group, but much higher than those in the C1 group. These results indicate that exogenous Mo application increased SOD activity and maintained stable contents of MDA, soluble proteins, and chlorophyll, and GPX activity in the rape plants of low Cd treatment. Despite relative changes in proline and soluble sugar contents, these parameters could not be recovered to the levels of the control group.

When the plants were treated with 6.0 mg/kg of Cd, both MDA and proline contents increased in the C2 group compared to the CK group, and decreased with increasing Mo concentration from 0 to 100 mg/kg (*p* < 0.05). The parameter values decreased by 52% and 12%, respectively (*p* < 0.05), in group C2M2 with 100 mg/kg Mo, as compared to group C2. There was a rebound in group C2M3 with 200 mg/kg Mo, and the parameter values were higher than in the CK group but lower than in the C2 group (*p* < 0.05). The contents of soluble proteins, soluble sugars, and chlorophyll all decreased in C2 compared to CK, and constantly increased with increasing Mo concentration (*p* < 0.05). The parameter values increased by 2.88-, 1.33-, and 1.19-fold, respectively (*p* < 0.05) in C2M3 with 200 mg/kg Mo, but were all lower than in the CK group. The activities of SOD and GPX decreased in C2 compared to CK, and increased with increasing Mo concentration from 0 to 100 mg/kg (*p* < 0.05). The enzyme activities increased by 1.19- and 1.35-fold, respectively, in C2M2 group with 100 mg/kg Mo (*p* < 0.05), but decreased in C2M3 with 200 mg/kg Mo (*p* < 0.05).

When different concentrations of exogenous Mo were applied alone, MDA and proline contents first decreased (M1–M2) and then increased (M3) with increasing Mo application compared to the CK group (*p* < 0.05). By contrast, the contents of soluble proteins, soluble sugars, and chlorophyll, as well as the activities of SOD and GPX, increased first (M1–M2) and then decreased (M3) with increasing Mo application (*p* < 0.05).

### 3.2. Effect of Mo on Chlorophyll Fluorescence Parameters in Cd-stressed Rape

**Figure 2:**
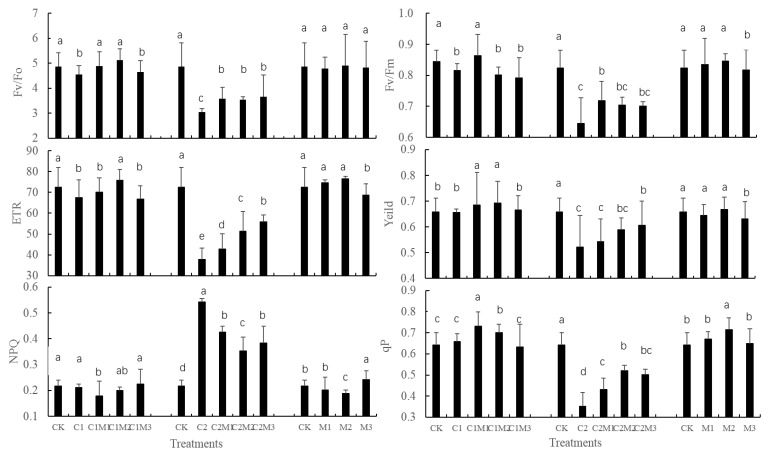
Effect of exogenous Mo on chlorophyll fluorescence parameters in rape plants under different levels of Cd treatments (*n* = 4). Note: Data are the mean ± standard deviation. Different lowercase letters above the error bars indicate significant differences (*p* < 0.05). CK (control group), C1 (treatment by 0.5 mg/kg Cd), C1M1 (treatment by 0.5 mg/kg Cd and 50 mg/kg Mo), C1M2 (treatment by 0.5 mg/kg Cd and 100 mg/kg Mo), C1M3 (treatment by 0.5 mg/kg Cd and 200 mg/kg Mo), C2 (treatment by 6.0 mg/kg Cd), C2M1 (treatment by 6.0 mg/kg Cd and 50 mg/kg Mo), C2M2 (treatment by 6.0 mg/kg Cd and 100 mg/kg Mo), C2M3 (treatment by 6.0 mg/kg Cd and 200 mg/kg Mo), M1 (treatment by 50 mg/kg Mo), M2 (treatment by 100 mg/kg Mo), M3 (treatment by 200 mg/kg Mo).

Figure 2 shows the effect of exogenous Mo on chlorophyll fluorescence parameters in rape plants under different levels of Cd. When the plants were treated with 0.5 mg/kg of Cd only, Fv/Fo, Fv/Fm, and ETR, all slightly decreased in the C1 group compared to the CK group (*p* < 0.05); yield, NPQ decreased and qP increased slightly, but not significantly. After combined Mo application, Fv/Fo, Fv/Fm, yield, ETR, and qP all increased first and then decreased with increasing Mo concentration, while NPQ exhibited an inverse trend in C1M1–C1M3 groups. The highest Fv/Fo, yield, and ETR were recorded in the C1M2 group with 100 mg/kg of Mo; this result means that when the soil was polluted by 0.5 mg/kg of Cd, the stress on these three parameters could be mitigated by applying 100 mg/kg of Mo. With regard to Fv/Fm, qP, and NPQ, these parameters were better improved in the C1M1 group with 50 mg/kg of Mo; this result also implies that Fv/Fo, yield, and ETR had more sensitive responses to Cd stress.

When the plants were treated with 6.0 mg/kg of Cd only, Fv/Fo, Fv/Fm, yield, ETR, and qP all decreased in the C2 group compared to the CK group (*p* < 0.05), while NPQ appeared to increase (*p* < 0.05). After combined Mo application, Fv/Fo, Fv/Fm, and qP all increased first and then decreased slightly with increasing Mo concentration, while an inverse trend was recorded for NPQ, and continuous increases occurred in yield and ETR. Among these, ETR, NPQ, and qP experienced the largest changes, followed by Fv/Fo; however, the parameter values remained much lower in C2M1–C2M3 groups than in the CK group (*p* < 0.05). The results demonstrate that 6.0 mg/kg Cd treatment caused substantial stress on chlorophyll fluorescence parameters in rape plants; the Cd stress was effectively mitigated by exogenous application of Mo, but the parameter values could not be recovered to the levels of the control group.

When 200 mg/kg of Mo was applied alone, all the chlorophyll fluorescence parameters relatively decreased in group M3 compared to CK (except for an increase in NPQ). This result means that 200 mg/kg of Mo exhibited a negative effect on plant physiology in rape.

### 3.3. Effect of Mo on the Uptake of Inorganic Cations in Cd-Stressed Rape

Table 3 summarizes the contents of inorganic cations in Cd-treated rape plants with or without exogenous application of Mo. When the plants were treated with 0.5 mg/kg of Cd only, K^+^, Na^+^, Ca^2+^, and Mg^2+^ contents all decreased slightly, but not significantly, in group C1 compared to CK. However, all cation contents increased with increasing Mo concentration and were higher in C1M1–C1M3 groups than in the CK group. The largest increase was recorded for K^+^ content (25.3%; *p* < 0.05), followed by those in Na^+^, Mg^+^, and Ca^2+^ contents (9.63%, 7.60%, and 3.09%, respectively).

When the plants were treated with 6 mg/kg of Cd only, K^+^ content decreased considerably in group C2 compared to CK (*p* < 0.05), but increased with increasing concentration of Mo compared to C2 (79.2%; *p* < 0.05). The single treatment with 6 mg/kg of Cd also decreased Na^+^, Ca^2+^, and Mg^2+^ contents, but their decreases were relatively small. Combined Mo application relatively increased Na^+^, Ca^2+^, and Mg^2+^ contents compared to the C2 group, yet Na^+^ and Ca^2+^ contents slightly decreased again in the C2M3 group with 200 mg/kg of Mo. Moreover, the cation contents (especially K^+^) increased in Cd-stressed rape plants with exogenous Mo, when compared to single Cd treatment. This result shows that Mo increased plant uptake of these inorganic cations in Cd-stressed rape, and exogenous application of Mo effectively reduced the inhibitory effect of Cd stress on K^+^, Na^+^, Ca^2+^, and Mg^2+^ uptake in rape plants.

### 3.4. Effect of Mo on Cd Uptake, Enrichment, and Translocation in Cd-Stressed Rape

Table 4 provides the Cd content in Cd-treated rape plants with or without exogenous application of Mo. The Cd uptake by the shoots and roots increased with increasing concentration of Cd treatment (*p* < 0.05), and the heavy metal content in Cd-treated plants was higher than in the CK group, especially under high Cd level (*p* < 0.05). When the plants were treated with 0.5 mg/kg of Cd, the Cd uptake by the shoots and roots increased and then decreased with increasing Mo concentration compared to the CK group (*p* < 0.05). The application of 200 mg/kg of Mo resulted in lower Cd uptake by the shoots and roots in the C1M3 group compared to the CK group, but not significantly (*p* < 0.05). With increasing Mo application, the enrichment factor of Cd in rape appeared to decrease gradually, while the translocation factor increased first and then decreased; the tolerance index increased gradually.

When the plants were treated with 6.0 mg/kg of Cd, both shoot and root Cd contents increased in the C2 group compared to the CK group (*p* < 0.05), and Cd content was higher in the roots than the shoots. Increasing application of Mo caused remarkable decreases in both shoot and root Cd contents of C2M1–C2M3 groups compared to the C2 group (*p* < 0.05). The bioconcentration factor of Cd exhibited a gradually decreasing trend with increasing Mo application, while the translocation factor decreased first and then increased; reversely, the tolerance index increased first and then decreased. Compared to single Cd treatment, combined Mo application reduced the bioconcentration factor and increased the tolerance index, but there was little change in the translocation factor. These results indicate that exogenous Mo effectively reduced Cd uptake while exhibiting little effect on Cd translocation in rape plants. The interaction between high concentrations of Mo and Cd considerably reduced the tolerance of rape to Cd.

## 4. Discussion

### 4.1. Exogenous Mo Benefits Plant Physiology and Biomass in Cd-Stressed Rape

Various biomass parameters can directly reflect the growth of plants and their stress level. This study showed that low Cd treatment inhibited length growth while promoting weight increase in rape plants; high Cd treatment inhibited both length growth and weight increase. The dose-dependent effect of Cd is consistent with previous results of Meng et al. [42] based on a study involving rape plants under Cd pollution. The biomass of Cd-stressed rape plants was significantly increased by applying 100 mg/kg of Mo. This effect could be attributed to the role of Mo in stabilizing plant physiological functions, facilitating vegetative growth, and binding and precipitating Cd [43].

Malondialdehyde content indicates the level of cell membrane damage and higher MDA accumulation indicates a weaker self-protection ability of the plant [44]. Free proline is an indicator of anti-stress response in plants, and its content rises rapidly under external stress [45]. In the present study, both 0.5 and 6 mg/kg of single Cd treatments induced increases in leaf MDA and proline contents in potted rape plants. A plausible reason is that Cd stress causes cellular damage and peroxidative loss of free radicals in rape leaves; these lead to intracellular lipid peroxidation of intracellular membranes and initiates the stress defense mechanisms, thereby causing the increases in MDA and proline contents in rape. The contents of MDA and proline decreased in rape leaves under combined Mo application, which proves that Mo can effectively reduce Cd-induced damage of intracellular membranes in rape [46,47].

The content of soluble proteins in a plant can indicate the presence of heavy metal stress. Under heavy metal stress, a high content of soluble proteins can protect the plant by reducing the osmotic potential of cells [48]. Additionally, plants in a stress state can adjust their soluble sugar content to reduce the harm of external stress, although the content of soluble sugars may decline in plants under long-term stress [49]. Here, the contents of soluble proteins and sugars significantly decreased in the leaves of rape plants under single Cd treatment compared to the control group; both parameters increased in the plants under combined Mo application, and soluble sugar content even reached the level of the control group. This result reveals that Mo can effectively improve the photosystem, stabilize the transportation channel of soluble sugars, and promote the production of soluble proteins in rape, thereby resisting Cd pollution. However, high Cd stress may destruct the photosynthetic system, which in turn causes over-decomposition of soluble proteins; the decomposition of insoluble sugars may also be impaired, preventing the replenishment of soluble sugar [50].

Chlorophyll is a green pigment for plant photosynthesis [51]. This study showed that single Cd treatment resulted in a decrease in chlorophyll content in the leaves of rape plants. Cd may exist in the cell wall of mesophyll cells, inhibiting chlorophyll synthesis in the leaves of rape [52,53]. By contrast, exogenous Mo increased chlorophyll content in the leaves of Cd-stressed rape. Indeed, Mo can stabilize the chloroplast structure, increase the number and volume of chloroplast grana, and enhance chlorophyll synthesis [54,55].

SOD and GPX are two important antioxidant enzymes, whose activities indicate the severity of plant damage caused by exogenous pollutants [56,57]. Here, SOD and GPX activities followed similar trends in the leaves of rape plants with the co-treatment of Mo and Cd. The activities of both enzymes were reduced by single Cd treatment, while they were enhanced by single Mo treatment. These results demonstrate that Cd can destroy the integrity of cell membrane structure by increasing the peroxidation of cell membrane lipids; Mo can effectively enhance the antioxidant capacity of rape and reduce the harm of Cd stress [58]. In particular, 6.0 mg/kg Cd treatment caused substantial stress on plant physiology in rape, and this stress was effectively inhibited by applying 50 or 100 mg/kg of exogenous Mo. An appropriate amount of exogenous Mo exhibits a positive effect on plant physiology, but excessive Mo would cause stress on the rape plants.

### 4.2. Exogenous Mo Improves Chlorophyll Fluorescence Parameters in Cd-Stressed Rape

Leaf chlorophyll fluorescence parameters, which indicate the photochemical activity and efficiency of PSII, are sensitive to external stress. For example, Fv/Fm represents the potential photosynthetic quantum yield of PSII, and Fv/Fo is indicative of the potential activity of PSII. Yield, namely the quantum yield in light adapted plants, indicates the actual light-harvesting efficiency at the PSII reaction center, while ETR represents the apparent electron transfer efficiency of PSII under actual light intensity [54]. Here, minor changes occurred in these four chlorophyll fluorescence parameters in the leaves of rape plants treated with 0.5 mg/kg Cd and Mo separately, whereas significant decreases in parameter values were observed in plants treated with 6.0 mg/kg Cd only, as compared to the control group. The possible mechanisms are as follows. The low level of Cd stress does not destruct the cell wall of mesophyll cells in rape plants; although chloroplasts are limited in capturing light and transmitting quantum, they could still perform photosynthesis. By contrast, the high level of Cd considerably reduces the activity and quantum yield of photosystem; a large amount of Cd may directly act on plant tissues of rape, deactivating protein complexes on the thylakoid membranes of chlorophylls, and thus causing partial photoinhibition at the PSII reaction center [59,60].

Combined Mo application increased Fv/Fo, Fv/Fm, yield, and ETR in the leaves of rape plants under two different levels of Cd pollution. This may be attributable to the function of Mo in plants; Mo can promote the synthesis of ascorbic acid and hence photosynthetic enzymes, thereby increasing the activity of PSII [61]. Here, the ability of mesophyll cells to harvest light was enhanced in Cd-stressed rape plants by applying Mo, which is consistent with the result of Kumchai et al. [62] reported in cabbage seedlings (*Brassica oleracea* L.) treated with Mo. The changes in rape plants in response to Cd stress under Mo application illustrate that rape can adjust the light-harvesting ability and electron transfer ability of PSII through regulation of Mo uptake, thereby minimizing the damage of PSII reaction center [63].

qP represents the photochemical quenching coefficient of plants under light conditions, while NPQ represents the non-photochemical quenching coefficient in plant systems during heat dissipation [64]. In the present study, rape plants processed excess light via photochemical quenching under 0.5 mg/kg Cd treatment with and without Mo application. By contrast, the excess light absorbed was quenched mainly via heat dissipation in the plants treated with 6.0 mg/kg Cd only. This result indicates that high Cd stress can impair the light conversion efficiency of PSII, and most of the light absorbed by natural pigments is used for heat dissipation at the PSII reaction center. Considering the quantum yield, the rape plants were seriously polluted by Cd in this study, and the light absorbed and used by the plants was derived mainly from the light adsorbed by antenna pigments. Moreover, qP increased while NPQ decreased with co-treatment of Mo and Cd compared to the control group. These changes may be attributable to the effect of Mo in promoting the synthesis of total free amino acids in the plant, which in turn improves leaf photosynthetic capacity [65,66].

### 4.3. Exogenous Mo Facilitates Cation Uptake in Cd-Stressed Rape

In plants, K^+^, Na^+^, Ca^2+^, and Mg^2+^ ions not only maintain the balance of potential energy, but are also components or key supporting elements of important functional enzymes [67]. Here, these cation contents all decreased in rape plants with increasing concentration of Cd treatment, especially under the high level of Cd (6 mg/kg). The results indicate an inhibitory effect of Cd on K^+^, Na^+^, Ca^2+^, and Mg^2+^ uptake in rape plants, and this effect was most pronounced for K^+^. A plausible reason for the intense response of K^+^ content to Cd stress is that Cd enters the rape plant through K^+^ ions, thereby reducing K^+^ uptake by the plant. The second most sensitive response was recorded for Ca^2+^ ions, which is in agreement with the study results of Zhang et al. [68] in water spinach (*Ipomoea aquatica* Forsk.). When treated with low Cd (0.5 mg/kg), combined Mo application significantly increased K^+^, Na^+^, Ca^2+^, and Mg^2+^ contents in rape plants. In the treatment with high Cd (6 mg/kg), the uptake of these cations increased with increasing Mo application, but their contents were still significantly lower than in the control group. This result demonstrates that Cd stress can destruct transport ion channels and relatively decrease the uptake of major ions in plants. Another study suggests that selenium (Se) can improve plant uptake of elements such as K, Ca, Mg, and Fe [69]. Similar to Se, here Mo was found to play a positive role in plant nutrient uptake.

### 4.4. Exogenous Mo Modulates Cd Uptake, Enrichment, and Translocation in Rape

The uptake of heavy metals can be reduced through various pathways in plants when subjected to heavy metal stress [70]; for example, the plants interact with the environment through the rhizosphere. However, a certain amount of heavy metals would accumulate in the plant upon long-term exposure to the environment polluted by heavy metals. In the present study, the Cd content in all rape plants treated with Cd exceeded the allowable limit for Cd in vegetables according to China’s National Standard for Maximum Levels of Contaminants in Foods (GB 2762-2017) [71]. This result is indicative of Cd pollution in the rape plants caused by the two different concentrations of Cd added to the pot soil. The high Cd content may also be related to the intrinsic ability of rape plants to enrich Cd. A study has reported on soil remediation using rape for Cd enrichment [72]. After combined Mo application, the Cd content in the rape plants of low Cd treatment significantly decreased with increasing Mo concentration. Notably, the Cd content in low Cd-treated plants with 200 mg/kg of Mo was lower than the allowable limit specified in China’s National Standard. Based on this result, Mo application can effectively reduce Cd content in rape plants when treated with a low level of Cd, and this finding is in accordance with the results of other physiological parameters and biomass of rape.

Here, exogenous Mo effectively reduced Cd intake in the rape plants when treated with high level of Cd, and the Cd content decreased significantly with increasing Mo concentration. However, due to excessively high levels of Cd content and the enrichment of Cd by rape itself, the results were still higher than the national standard [71]. Moreover, rape showed high capacities for Cd enrichment and translocation, indicating that Cd is an active metal [73]. The application of Mo reduced the enrichment capacity of Cd in rape by decreasing Cd uptake, but it had little effect on the translocation capacity of Cd. A plausible reason is that Mo can chemically react with Cd in the soil and thereby reduce Cd uptake in rape plants; however, it cannot reduce the translocation capacity of Cd that has entered the rape plant. The possible mechanism of Mo is different from the effect of Se on heavy metals entering the plant [67,74]. Furthermore, this study showed that the tolerance index of rape was significantly reduced by Cd treatment. This could be explained by the fact that Mo promotes the synthesis of photosynthesis-related enzymes and the photosynthetic cycle in the plant [75], thereby reducing the toxicity of Cd entering the plant of rape.

## 5. Conclusions

Based on pot experiment, this study indicated that soil pollution by a low concentration of Cd had little effect on the physiological parameters, biomass, photosynthetic parameters, and cation uptake in rape plants, but it promoted weight increase in the shoots of rape. By contrast, a high concentration of Cd pollution exhibited a significant negative effect on the physiology, biomass, photosynthesis, and cation uptake in rape. The toxicity of Cd to the physiology and morphology of rape plants was significantly mitigated by applying exogenous Mo to the soil. The mitigation effect of Mo could be achieved through the following mechanism. Mo participated in physiological reactions to promote the synthesis of photosynthesis-related enzymes and the photosynthetic cycle in the rape plant, thereby reducing the toxicity of Cd entering the plant. The application of 100 mg/kg of Mo could effectively improve the physiological activities and biomass while preventing the Cd uptake in rape under the experimental conditions.

## Figures and Tables

**Table 1:** Experimental design of heavy metal concentrations per kg of soil.

Treatment	Cd (mg)	Mo (mg)	Treatment	Cd (mg)	Mo (mg)
CK	0	0	C2M1	6	50
C1	0.5	0	C2M2	6	100
C1M1	0.5	50	C2M3	6	200
C1M2	0.5	100	M1	0	50
C1M3	0.5	200	M2	0	100
C2	6	0	M3	0	200

Note: The dosages of reagents added were converted to the amounts of Cd and Mo.

**Table 2:** Changes in leaf physiological parameters of rape plants with the co-treatment of Mo and Cd (*n* = 4).

Treatment	MDA	Proline	Soluble Proteins	Soluble Sugars	Chlorophyll	SOD	GPX
	(mmol/kgFW)	(mg/kgFw)	(mg/kg·10^3^)	(mg/kg·10^3^)	(mg/kg·10^3^)	(U/g·h)	(U/g·h)
CK	14.6 ± 0.8bc	95.2 ± 6.4c	17.4 ± 1.2a	66.2 ± 2.2a	2.51 ± 0.12a	260 ± 12b	97.5 ± 8.9a
C1	19.7 ± 1.6a	142 ± 5a	15.8 ± 1.1b	59.4 ± 2.1b	2.14 ± 0.31c	251 ± 15c	71.2 ± 3.6c
C1M1	15.4 ± 2.2b	125 ± 8ab	14.2 ± 1.7bc	58.1 ± 2.1b	2.25 ± 0.13b	256 ± 15b	80.8 ± 7.3b
C1M2	13.7 ± 0.7c	115 ± 4b	17.5 ± 0.8a	60.2 ± 1.3b	2.39 ± 0.15b	277 ± 18a	85.8 ± 6.7ab
C1M3	12.8 ± 1.8c	121 ± 6ab	18.7 ± 0.9a	57.2 ± 2.1b	2.58 ± 0.31a	242 ± 9c	68.7 ± 2.2c
CK	14.6 ± 0.8c	95.2 ± 6.4c	17.4 ± 1.2a	66.2 ± 2.2a	2.51 ± 0.12a	260 ± 12a	97.5 ± 8.9a
C2	42.7 ± 1.5a	211 ± 25a	4.3 ± 0.5c	40.1 ± 1.7c	1.73 ± 0.10c	204 ± 28b	40.4 ± 7.7bc
C2M1	39.7 ± 1.9a	221 ± 27a	6.4 ± 0.1c	47.2 ± 1.5bc	1.78 ± 0.24c	228 ± 22ab	45.1 ± 2.9bc
C2M2	25.7 ± 2.1b	187 ± 14b	9.1 ± 1.0bc	52.4 ± 1.9b	1.97 ± 0.15b	242 ± 20a	54.7 ± 3.7b
C2M3	38.7 ± 1.6a	212 ± 12a	12.4 ± 1.3b	53.2 ± 2.5b	2.06 ± 0.27b	210 ± 18b	35.5 ± 5.7c
CK	14.6 ± 0.8b	95.2 ± 6.4b	17.4 ± 1.2b	66.2 ± 2.2ab	2.51 ± 0.12ab	260 ± 12a	97.5 ± 8.9a
M1	10.4 ± 1.9c	92.3 ± 5.8b	20.4 ± 1.3a	68.1 ± 2.2a	2.63 ± 0.19a	270 ± 9a	98.8 ± 2.8a
M2	13.7 ± 2.1b	94.6 ± 8.5b	22.6 ± 0.8a	72.5 ± 1.9a	2.70 ± 0.11a	278 ± 19a	102 ± 13a
M3	21.7 ± 0.8a	109 ± 10a	13.7 ± 1.1c	54.4 ± 3.2b	2.33 ± 0.30b	232 ± 21b	88.0 ± 5.9b

Note: MDA = Malondialdehyde, SOD = Superoxide dismutase, GPX = Glutathione peroxidase. Data are the mean ± standard deviation. Values in the same column followed by different lowercase letters are significantly different (*p* < 0.05). CK (control group), C1 (treatment by 0.5 mg/kg Cd), C1M1 (treatment by 0.5 mg/kg Cd and 50 mg/kg Mo), C1M2 (treatment by 0.5 mg/kg Cd and 100 mg/kg Mo), C1M3 (treatment by 0.5 mg/kg Cd and 200 mg/kg Mo), C2 (treatment by 6.0 mg/kg Cd), C2M1 (treatment by 6.0 mg/kg Cd and 50 mg/kg Mo), C2M2 (treatment by 6.0 mg/kg Cd and 100 mg/kg Mo), C2M3 (treatment by 6.0 mg/kg Cd and 200 mg/kg Mo), M1 (treatment by 50 mg/kg Mo), M2 (treatment by 100 mg/kg Mo), M3 (treatment by 200 mg/kg Mo).

**Table 3:** The changes of inorganic cation contents in rape with the co-treatment of Mo and Cd (*n* = 4).

Treatment	K^+^	Na^+^	Ca^2+^	Mg^2+^
	(mg/kg)	(mg/kg)	(mg/kg)	(mg/kg)
CK	31.2 ± 2.2b	4.78 ± 0.12a	8.45 ± 0.25a	4.31 ± 0.12a
C1	29.7 ± 1.7b	4.36 ± 0.12a	8.41 ± 0.18a	4.21 ± 0.13a
C1M1	31.8 ± 2.5b	4.55 ± 0.23a	8.51 ± 0.26a	4.31 ± 0.14a
C1M2	35.7 ± 1.2a	4.75 ± 0.13a	8.66 ± 0.23a	4.44 ± 0.24a
C1M3	37.2 ± 1.6a	4.78 ± 0.22a	8.67 ± 0.07a	4.53 ± 0.27a
CK	31.2 ± 2.2a	4.78 ± 0.12a	8.45 ± 0.25a	4.31 ± 0.12a
C2	14.9 ± 1.8d	4.01 ± 0.17a	8.01 ± 0.19a	3.85 ± 0.28ab
C2M1	19.5 ± 0.9c	4.25 ± 0.22a	7.91 ± 0.22a	3.54 ± 0.16b
C2M2	22.5 ± 1.3bc	4.29 ± 0.37a	8.01 ± 0.14a	3.67 ± 0.25b
C2M3	26.7 ± 1.5b	4.18 ± 0.11a	7.96 ± 0.16a	3.95 ± 0.35ab
CK	31.2 ± 2.2b	4.78 ± 0.12a	8.45 ± 0.25a	4.31 ± 0.12a
M1	32.2 ± 0.5b	4.86 ± 0.23a	8.67 ± 0.16a	4.36 ± 0.14a
M2	37.7 ± 1.2a	4.92 ± 0.13a	8.76 ± 0.13a	4.49 ± 0.15a
M3	39.2 ± 1.6a	4.71 ± 0.22a	8.72 ± 0.27a	4.57 ± 0.18a

Note: Data are the mean ± standard deviation. Values in the same column followed by different lowercase letters are significantly different (*p* < 0.05). CK (control group), C1 (treatment by 0.5 mg/kg Cd), C1M1 (treatment by 0.5 mg/kg Cd and 50 mg/kg Mo), C1M2 (treatment by 0.5 mg/kg Cd and 100 mg/kg Mo), C1M3 (treatment by 0.5 mg/kg Cd and 200 mg/kg Mo), C2 (treatment by 6.0 mg/kg Cd), C2M1 (treatment by 6.0 mg/kg Cd and 50 mg/kg Mo), C2M2 (treatment by 6.0 mg/kg Cd and 100 mg/kg Mo), C2M3 (treatment by 6.0 mg/kg Cd and 200 mg/kg Mo), M1 (treatment by 50 mg/kg Mo), M2 (treatment by 100 mg/kg Mo), M3 (treatment by 200 mg/kg Mo).

**Table 4:** The changes of Cd content in rape plants with the co-treatment of Mo and Cd (*n* = 4).

Treatment	Cd (mg/kg)		Bioconcentration	Translocation Factor	Tolerance Index (%)
	Shoot	Root	Factor		
CK	0.032 ± 0.009b	0.074 ± 0.046c	0.442	0.432	100
C1	0.104 ± 0.033a	0.222 ± 0.014a	0.441	0.468	96.4
C1M1	0.094 ± 0.021a	0.201 ± 0.014a	0.412	0.517	103
C1M2	0.044 ± 0.012b	0.101 ± 0.024b	0.196	0.436	113
C1M3	0.024 ± 0.008b	0.066 ± 0.015c	0.122	0.364	124
CK	0.032 ± 0.009d	0.074 ± 0.046d	0.442	0.432	100
C2	0.657 ± 0.052a	1.09 ± 0.07a	0.28	0.603	82.4
C2M1	0.386 ± 0.025b	0.754 ± 0.042b	0.183	0.512	89.3
C2M2	0.297 ± 0.024b	0.601 ± 0.015b	0.144	0.494	92.9
C2M3	0.191 ± 0.012c	0.354 ± 0.034c	0.091	0.54	85.0

Note: For Cd content, data are the mean ± standard deviation. Values in the same column followed by different lowercase letters are significantly different (*p* < 0.05). CK (control group), C1 (treatment by 0.5 mg/kg Cd), C1M1 (treatment by 0.5 mg/kg Cd and 50 mg/kg Mo), C1M2 (treatment by 0.5 mg/kg Cd and 100 mg/kg Mo), C1M3 (treatment by 0.5 mg/kg Cd and 200 mg/kg Mo), C2 (treatment by 6.0 mg/kg Cd), C2M1 (treatment by 6.0 mg/kg Cd and 50 mg/kg Mo), C2M2 (treatment by 6.0 mg/kg Cd and 100 mg/kg Mo), C2M3 (treatment by 6.0 mg/kg Cd and 200 mg/kg Mo).
